# Addendum: Primary care management of the coronavirus (COVID-19)

**DOI:** 10.4102/safp.v62i1.5144

**Published:** 2020-06-10

**Authors:** Bob Mash

**Affiliations:** 1 Division of Family Medicine and Primary Care, Faculty of Medicine and Health Sciences, University of Stellenbosch, Cape Town, South Africa

Since writing this CPD article (https://doi.org/10.4102/safp.v62i1.5115), there have been a few changes in the guidelines for primary care management and some new information on COVID-19 (coronavirus infectious disease - 2019).

The case definition now no longer requires additional risk factors (close contact, travel history or exposure at a health facility) with the typical symptoms. Persons with acute respiratory illness with the sudden onset of at least one of the following: cough, sore throat, shortness of breath or fever (≥ 38 °C [measured] or history of fever [subjective]) should be investigated for COVID-19.

It has become apparent that, in addition to the symptoms listed in the article, a number of people also have a loss of taste and smell.

It has also become clear that the virus can be transmitted by asymptomatic people, often in the days before they develop symptoms.

There has been an active debate on the value of masks and the South African government is recommending that everyone wear a cloth mask when outside the home. Surgical and N95 masks should be used according to the guidelines and reserved for those who need them.

Although the provision of tests, to prove that the person is no longer infectious, is ideal, it is not feasible in the majority of South Africa as a result of the current testing capacity. Patients are therefore declared recovered once they have completed 14 days of isolation and are symptom-free. Timeframes for the period of isolation are as follows:

- Asymptomatic patients: 14 days from time of positive test
- Mild disease: 14 days from onset of symptoms
- Moderate or severe disease: 14 days following clinical stabilisation (no longer requiring oxygen)

It has become clear that a number of people develop ‘silent hypoxia’. In other words, they may not appear to be short of breath, but are in fact hypoxic with a low oxygen saturation. Checking oxygen saturation is therefore a useful part of the assessment. Those with an oxygen saturation < 95%, a respiratory rate > 25/minute or who are short of breath should be offered oxygen.

Refer to hospital those with more than a mild disease and if possible initiate treatment. Oxygen can be given by nasal prongs (1 L/min – 4 L/min) or face mask (6 L/min – 10 L/min) and, if oxygen saturation remains below 90%, a non-rebreather reservoir bag can be added to the face mask and oxygen given at 10 L/min – 15 L/min. Manage in the prone position if possible.

If blood pressure (BP) is < 90/60mmHg then consider giving 250 ml sodium chloride intravenous slowly over 30 min. Repeat until systolic BP is > 90 mmHg. Stop if breathing worsens.

If severe bacterial pneumonia is part of the differential diagnosis, give ceftriaxone 1 g intravenous/intramuscular and azithromycin 1 g orally.

Treat co-morbidities (asthma, chronic obstructive pulmonary disease [COPD], diabetes, cardiac failure) as necessary. If the patient has asthma or COPD and requires inhaled treatment, this should be given via a spacer and not a nebuliser to avoid creating an aerosol of COVID-19.

